# Perceived effects of health status on sexual activity in patients with axial spondyloarthritis: a 5-year follow-up study

**DOI:** 10.1007/s00296-024-05758-3

**Published:** 2024-12-28

**Authors:** Gudrun Rohde, Kari Hansen Berg, Are Hugo Pripp, Glenn Haugeberg

**Affiliations:** 1 Faculty of Health and Sport Sciences, University of Agder, Servicebox 422, 4604 Kristiansand, Norway; 2 Research Unit, Sorlandet Hospital, Kristiansand, Norway; 3 Oslo Centre of Biostatistics and Epidemiology, Research Support Services, Oslo University Hospital, Oslo, Norway; 4 Faculty of Health Sciences, Oslo Metropolitan University, Oslo, Norway; 5 Division of Rheumatology, Department of Internal Medicine, Sorlandet Hospital, Kristiansand, Norway; 6 Department of Neuromedicine and Movement Science, Faculty of Medicine and Health Sciences, Norwegian University of Science and Technology, Trondheim, Norway

**Keywords:** Axial spondyloarthritis, Health status, Sexual activity, Follow-up

## Abstract

Axial spondyloarthritis (ax-SpA) causes pain, fatigue, stiffness, loss of physical function, and poor health status, which can influence sexual activity and enjoyment. To explore whether patients with ax-SpA perceive that their health status effects their sexual activity and to identify predictors of these perceived effects on sexual activity after a 5-year follow-up. Data about demographics, disease, medication, health-related quality of life (HRQOL), and sexual quality of life (SQOL) were collected at the baseline and 5-year follow-up. The perceived effect of health status on sexual activity was measured by question 15 in the 15D questionnaire. Data were analysed using the McNemar and independent paired *t* tests and logistic regression. In the 244 patients with ax-SpA (30% women, 70% men; mean age, 46 years), measures reflecting disease activity decreased and comorbidities increased, and more patients were treated with biological drugs at 5 years. Compared with patients whose health status had little/no effect on sexual activity (n = 200), those who perceived that their health status had a large effect on sexual activity (n = 44) were older, exercised less, fewer were employed, had more comorbidities, higher disease activity, and lower HRQOL and SQOL. The baseline predictors of a negative effect of health status on sexual activity were old age and low SQOL. Patients reporting that their health status had a large effect on sexual activity at 5 years were older, had more disease activity, and lower HRQOL and SQOL.

## Introduction

Axial spondyloarthritis (ax-SpA) is a chronic inflammatory disorder of the axial skeleton that causes pain, fatigue, stiffness, and loss of physical function. The burden of disease has been reported by patients to affect their lives [1, 2]. All of these effects can influence sexual activity and enjoyment [3–5] and may be associated with impaired health-related quality of life (HRQOL) and sexual quality of life (SQOL) [6, 7]. HRQOL is a subjective and multidimensional concept that is defined as an individual’s experience of their general health status, including their physical, social, and mental well-being [8]. SQOL is not clearly defined in the literature, but it includes the relationship between sexual dysfunction and HRQOL [9, 10]. However, better disease control and a treat to target approach of the patients might imply less pain and better physical function and thereby influence HRQOL, SQOL and sexual activity positively [3].

Sexual activity is considered to be an important part of sexual health, health, HRQOL and SQOL. Sexual activity is influenced by personal characteristics, interpersonal relationships, family circumstances, socio-cultural conditions, environment, physical and mental health and hormonal status [11]. Furthermore, physical difficulties [12–14], fatigue, and sleep disturbance [15], can influence sexual activity negatively. The frequencies of sexual activity and level of intimacy might change in different phases of life, decreasing with ager. Moreover, sexual activity might be affected by a person’s view of their body, having a partner, culture and society [16].

Previous studies have explored the impacts of ax-SpA on sexuality mainly by assessing sexual dysfunction or sexual problems in cross-sectional studies [17–20] and there are limited data about how the perceptions of health status influence sexual activity in patients with ax-SpA. Baseline findings for a cross-sectional study of the cohort described here identified associations of self-reported negative effects on sexual activity with female gender, high body mass index (BMI), current smoking, and low HRQOL [20]. We have also identified that impaired SQOL is associated with the baseline variables female sex, high BMI, measures reflecting disease activity, and current use of biologic disease-modifying anti-rheumatic drugs (bDMARDs) [21]. Another study has reported no changes in SQOL after 5 years [22].

Furthermore, previous studies have also shown that patients with ax-SpA report HRQOL scores that are similar to those for patients with other inflammatory diseases [4, 23] but lower than those in healthy controls [3, 5, 24–26]. Women with ax-SpA report lower HRQOL than men [27–29]. Low HRQOL in patients with ax-SpA has been found to be associated with fatigue [30], increased disease activity, low amounts of daily activity and exercise [26, 31–33], pain, and adverse psychological factors, such as body image disturbance, anxiety, and depression [34, 35]. Better disease control and maintenance of HRQOL over years have been reported more recently [6].

Previous studies in patients with ax-SpA have focused on sexual dysfunction, sexual problems, HRQOL and SQOL. To our knowledge, no studies have explored the perceived effects of health status on sexual activity from the long-term perspective in patients with ax-SpA. Therefore, the aims of this study were to explore these long-term effects and to identify predictors associated with the perceived effects of health status on sexual activity in patients with ax-SpA followed for 5 years.

## Methods

### Patient recruitment

Patients with ax-SpA were recruited consecutively from two public outpatient rheumatology clinics in southern Norway: Martina Hansens Hospital (MHH) and Sørlandet Hospital (SSHF). We have previously reported the baseline data from a prospective study of this cohort and details of the recruitment process [7, 26]. For inclusion, the patients had to be 18 years or older, met the Assessment of Spondyloarthritis International Society (ASAS) criteria for ax-SpA, mentally capable to give informed consent and able to read and write Norwegian. Exclusion criteria were few; Uncapable of giving informed consent and unable to read and write Norwegian [36]. Only a small number of patients fulfilling the inclusion criteria choose not to take part (4 at MHH and 4 at SSHF).

## Data collection

The same data collected at the baseline were also collected at the 5-year follow-up and included demographic and disease- and treatment-related variables, as described previously [26]. Because of funding restrictions, the most recently included patients at MHH were not invited for a follow-up examination. Among the 380 patients with ax-SpA examined at the baseline (MHH, n = 252; SSHF, n = 128), 244 patients (MHH, 53%; SSHF, 84%) were re-examined after 5 years. The patients who did not participate in the 5-year follow-up data collection had significantly more comorbidities (0.93 [1.05] vs. 0.58 [0.81], respectively; *p* = 0.001) at the baseline than those included in the follow-up study. For other variables, there were no significant differences between participants and non-participants at the 5-year follow-up (data not shown).

Data were collected from interviews, questionnaires, laboratory tests, and physical examinations. The demographic data collected by interviews included age, gender, education level (< 11 years, 11–13 years, and > 13 years), work status (employed), physical exercise (exercise for ≥ 1 h per week vs. < 1 h per week), BMI (kg/m^2^), and current smoking. Disease duration was defined as the time between the date when the patients fulfilled the ASAS criteria for ax-SpA and the date of their inclusion in the study. Data on human leucocyte antigen (HLA) 27 status was collected. Disease activity was assessed using the Bath Ankylosing Spondylitis Activity Index (BASDAI) (range, 1–10), Maastricht Ankylosing Spondylitis Enthesitis Score (MASES) (range, 1–13), and C-reactive protein (CRP) concentration (mg/dl). Physical function was assessed using the Bath Ankylosing Spondylitis Functional Index (BASFI) (range, 0–10) and the Health Assessment Questionnaire (HAQ) (range, 0–3) [37]. Data were also collected using the Bath Ankylosing Spondylitis Patient Global Score (BAS-G) (range, 0–10). Current medications including the use of non-steroidal anti-inflammatory drugs **(**NSAIDs), synthetic DMARDs (sDMARDs), bDMARDs, and prednisolone were recorded. Finally, data on comorbidities (yes/no) including heart, pulmonary, neurological, endocrine, haematological, gastrointestinal, urogenital, or other rheumatological diseases, mental disorders, and cancer were collected. A summed score was generated to reflect overall comorbidity. This score has been used in other studies [7, 20, 38, 39].

## Questionnaires

Item 15 in the 15D questionnaire was used to study the effects of health status on sexual activity at both the baseline and 5-year follow-up, and was used as the dependent variable in the statistical analyses [40]. Item 15 addresses the effects of health on sexual activity with the following response options:

My state of health:

1. ... has no adverse effect on my sexual activity.
2. ... has a slight effect on my sexual activity.
3. ... has a considerable effect on my sexual activity.
4. ... makes sexual activity almost impossible.
5. ... makes sexual activity impossible.

To analyse the effects of health on sexual activity, we dichotomized the five responses to item 15 in the 15D instrument that are related to sexual activity as reported in previous studies [20, 40, 41]. Responses 1 and 2 were grouped into ‘little/no effect’, and the other three categories were grouped into ‘large effects’. The entire 15D was used to measure HRQOL. The 15D questionnaire is a generic multidimensional standardized tool for evaluating HRQOL. This instrument is used primarily as a single index measure, but it can also be used as a profile utility measure. The 15D questionnaire captures the health status by assessing 15 dimensions: mobility, vision, hearing, breathing, sleeping, eating, speech, elimination, usual activities, mental function, discomfort and symptoms, depression, distress, vitality, and sexual activity [42]. Each dimension is assessed by one question with five response categories. The 15 D was translated into Norwegian from an English version independently by physicians, who discussed the translations and agreed on a consensus version. The Norwegian version had also been compared with the original Finnish version [43]. The questionnaire has been tested for psychometric properties in other studies in several countries including Norway [42, 43]. Furthermore, item 15 in the 15D questionnaire has been used in different patient groups focusing the perceive effect of health status on sexual activity [40, 41].

We also assessed HRQOL using the 36-item Short Form Survey Instrument (SF-36) to identify any differences in HRQOL between patients reporting that their health status has a large negative effect on their sexual activity versus those reporting little/no effect. The SF-36 is a self-reported generic questionnaire with eight domains: general health, bodily pain, physical functioning, role limitations (physical), mental health, vitality, social functioning, and role limitations (emotional). These domains can be combined into a physical and mental sum scale that reflects physical and mental health. The physical component summary (PCS) and mental component summary (MCS) scales were used in this study. For incomplete questionnaires, missing values were substituted using the scale instructions given by the developers of the questionnaire [44, 45]. The SF-36 scales were scored according to published scoring procedures, and each was expressed as a value from 0 to 100 where 100 represented excellent health [44, 45]. The questionnaire has been thoroughly tested for psychometric properties in other studies in several countries including Norway [44–47].

SQOL was assessed using the Sexual Quality of Life-Female (SQOL-F) questionnaire. The SQOL-F is a generic self-reported questionnaire and is used to assess the relationship between female sexual dysfunction and QOL [48]. The questionnaire can also be used for partners and male partners with minor modifications [10]. The SQOL-F comprises 18 items that have six possible responses: completely agree, moderately agree, slightly agree, slightly disagree, moderately disagree, and completely disagree. A total sum score for SQOL and for the SQOL subcategories of psychosexual feelings, sexual and relationship satisfaction, self-worthlessness, and sexual repression was calculated [49]. A higher score indicates better SQOL, except for sexual and relationship satisfaction, for which a low score indicates better SQOL [21].

## Statistical analyses

Statistical analyses were performed using IBM SPSS Statistics (v. 28; IBM Corp., Armonk, NY, USA). Continuous variables are presented as mean and standard deviation (SD) and categorical variables as number and percentage (%). Data distributions were checked using histogram for continuous data. The continuous data were considered normally distributed. To receive a better overview of categorical data, count and percentages were calculated. For comparison between groups, the chi-square test was used for categorical variables and the independent *t* test for continuous variables. The paired-sample *t* test and McNemar test were used to identify differences between the baseline and 5-year follow-up.

Multivariate associations with a negative effect of health status on sexual activity (question 15 in the 15D questionnaire) were explored using logistic regression analyses (block-wise procedure). The independent variables assessed at the baseline were chosen because of their clinical significance [50]. The independent variables were included as follows: in block 1, age, gender, living together/alone, education, and exercise; block, 2 BASDAI, BASMI, HAQ, and CRP; block 3, comorbidities; block 4, current medication; block 5, SQOL; and block 6, HRQOL. The final model included all independent variables from all blocks. The model fit was assessed using the Nagelkerke R square. The level of significance was set at *p* < 0.05.

## Ethical and legal aspects

The study was approved by the Regional Committee for Medical Research Ethics (REK #4.2007.2152).

## Results

### Demographic and disease-related characteristics

The recorded variables and comparisons between the baseline and 5-year follow-up data for the 244 patients with valid responses to question 15 in the 15D questionnaire at the 5-year follow-up are presented in Table 1. At baseline mean disease duration was 14 years (SD = 11). At the 5-year follow-up, fewer patients were smokers (42 [17%] vs. 66 [27%]; *p* < 0.001), fewer patients were employed (166 [69%] vs. 178 [75%]; *p* = 0.028), and more patients were using bDMARDs (86 [35%] vs. 57 [23%]; *p* = 0.001). At the 5-year follow-up, patients had significantly more comorbidities (1.0 [1.1] vs. 0.6 [0.8]; *p* < 0.001), lower CRP concentration (5.6 mg/dl [10.5] vs. 8.50 mg/dl [12.7]; *p* = 0.001), and better scores on the MASES (1.3 [2.4] vs. 3.4 [3.9]; *p* < 0.001), BAS-G (3.1 [2.6] vs. 3.8 [2.6]; *p* = 0.003), and HAQ (0.44 [0.43] vs. 0.55 [0.48]; *p* = 0.026).

**Table 1:** Comparisons between baseline and 5-year follow-up demographic data, disease markers, disease activity measures, damage, health status, comorbidities, and medications in 244 patients with axial spondyloarthritis

	Baseline	5-year follow-up	*p* value
Demographics			
Age, years	46.0 (10.9)		
Woman	74 (30%)	74 (30%)	
Man	170 (70%)	170 (70%)	
Disease duration (years)	14 (11)		
Married/cohabiting	190 (78%)	186 (76%)	0.728
Current smoker	66 (27%)	42 (18%)	< 0.001
Employed	178 (75%)	166 (69%)	0.028
Exercise ≥ 1 h/week	214 (88%)	217 (89%)	0.743
BMI (kg/m^2^)	27.0 (4.4)	27.1 (4.4)	0.568
Education			0.339
< 13 years	110 (45%)	105 (44%)	
≥ 13 years	133 (55%)	136 (56%)	
Comorbidity			
Mean total score for comorbidity (range, 0–10)	0.6 (0.8)	1.0 (1.4)	< 0.001
Disease activity measures			
CRP (mg/dl)	8.5 (12.7)	5.6 (10.5)	0.001
BASDAI	3.1 (2.1)	2.8 (2.2)	0.153
MASES	3.3 (3.1)	1.34 (2.4)	< 0.001
Damage			
BASMI	2.3 (2.0)	2.3 (2.1)	0.700
Health status			
BASFI	2.7 (2.2)	2.3 (2.2)	0.130
BAS-G	3.8 (2.6)	3.1 (2.6)	0.003
HAQ (range, 0–3)	0.55 (0.48)	0.44 (0.43)	0.026
Current treatment			
NSAIDs	98 (40%)	82 (34%)	0.105
Synthetic conventional DMARDs	15 (6%)	14 (6%)	1.00
Biologic DMARDs	57 (23%)	86 (35%)	0.001

Categorical data are presented as number (%) and continuous variables as mean (SD)

The McNemar test was used to compare categorical variables and paired-sample *t* test for continuous variables. Disease duration defined as the time between the date when the patients fulfilled the ASAS criteria for ax-SpA and inclusion date. *BMI* body mass index, *CRP* C-reactive protein, *BASDAI* bath ankylosing spondylitis disease activity index (range, 1–10), *MASES* the maastricht ankylosing spondylitis enthesitis score (range, 1–13), *BASMI* bath ankylosing spondylitis metrology index, *BASFI* bath ankylosing spondylitis functional Index (range, 0–10), *BAS-G* bath ankylosing spondylitis patient global score (range, 0–10), *HAQ* health assessment questionnaire (range, 0–3), *NSAIDs* non-steroidal anti-inflammatory drugs), *DMARDs* disease-modifying anti-rheumatic drugs

## Health status having no/little effects versus large effects on sexual activity

At the 5-year follow-up 44 (18%) patients reported that their health status had a large effect on their sexual activity, and 200 (82%) reported little/no effect. From the baseline to the 5-year follow-up, 24 patients changed their perception from a large effect to little/no effect, and 26 patients changed from little/no effect to a large effect.

A similar pattern was seen at both the baseline and 5-year follow-up for the comparison of patients perceptions of whether health status had little/no effect versus a large effect on sexual activity. However, the differences seemed to be larger at the 5-year follow-up (Table 2). At the 5-year follow-up, patients reporting that their health status had a large effect on sexual activity were older (54 [10] years vs. 50 [11] years; *p* = 0.014), fewer were employed (74% vs. 47%; *p* < 0.001), and exercised less (77% vs. 92%; *p* = 0.003). These patients also had more comorbidities (1.5 [1.3] vs. 0.8 [1.1]; *p* = 0.002), higher CRP concentration (10.2 [19.1] vs. 4.6 [7.0]; *p* = 0.002), higher BASDAI (4.7 [2.2] vs. 2.5 [2.0]; *p* < 0.001), higher BASFI (3.9 [2.2] vs. 2.0 [2.0]; *p* < 0.001), higher BAS-G (5.3 [2.6] vs. 2.6 [2.4]; *p* < 0.001), and higher HAQ (0.81 [0.45] vs. 0.37 [0.4]; *p* < 0.001).

**Table 2:** Comparing baseline and 5-year follow-up demographic data, disease markers, disease activity measures, damage, health status and comorbidity in 200 patients with axial spondyloarthritis responding their health status to have little/no effects on their sexual activity versus 44 patients reporting large effects

	Little/no effects baseline	Large effects baseline	*p* value	Little/no effects 5 years	Large effects 5 years	*p* value
Demographics						
Age, years	45.6 (10.9)	47.6 (11.2)	0.291	45.2 (10.9)	49.5 (10.1)	0.017
Women	61 (30%)	13 (71%)	0.901	31 (71%)	13 (29%)	0.901
Men	139 (70%)	31 (29%)		139 (69%)	61 (31%)	
Married/cohabiting	151 (76%)	39 (87%)	0.064	152 (77%)	34 (79%)	0.786
Current smoker	49 (25%)	17 (38%)	0.056	31 (17%)	11 (27%)	0.121
Employed	150 (78%)	28 (64%)	0.051	146 (74%)	20 (47%)	< 0.001
Exercise ≥ 1 h/week	179 (90%)	35 (80%)	0.054	183 (92%)	34 (77%)	0.003
BMI (kg/m^2^)	26.9 (4.2)	28.0 (5,1)	0.195	27.0 (4.2)	27.9 (5.5)	0.371
Education						
< 13 years	89 (45%)	21 (48%)	0.717	89 (45%)	21 (48%)	0.717
≥ 13 years	110 (55%)	23 (52%)		110 (55%)	23 /52%)	
Comorbidity						
Mean total score for comorbidity (range, 0–10)	0.5 (0.8)	0.8 (0.9)	0.062	0.8 (1.1)	1.5 (1.3)	0.002
Disease activity measures						
CRP (mg/dl)	8.5 (12.0)	8.6 (15.7)	0.973	4.6 (7.0)	10.2 (19.1)	0.002
BASDAI	3.0 (2.1)	4.0 (2.0)	0.004	2.5 (2.0)	4.7 (2.2)	< 0.001
MASES	2.9 (3.7)	4.8 (4.1)	0.007	1.2 (2.2)	1.5 (2.2)	0.310
Damage						
BASMI	2.3 (2.1)	2.4 (1.9)	0.820	2.3 (2.1)	2.6 (1.7)	0.421
Health status						
BASFI	2.5 (2.1)	3.8 (2.2)	0.001	2.0 (2.0)	3.9 (2.2)	< 0.001
BAS-G	3.5 (2.5)	5.1 (2.5)	< 0.001	2.6 (2.4)	5.3 (2.6)	< 0.001
HAQ (range, 0–3)	0.49 (0.45)	0.80 (0.52)	0.001	0.37 (0.40)	0.81 (0.45)	< 0.001
Current treatment						
NSAIDs	77 (39%)	21 (48%)	0.258	63 (32%)	19 (43%)	0.137
Synthetic conventional DMARDs	11 (6%)	4 (9%)	0.369	11 (6%)	3 (7%)	0.734
Biologic DMARDs	43 (22%)	14 (32%)	0.143	73 (37%)	13 (30%)	0.382

Categorical data are presented as number (%) and continuous variables as mean (SD)

Chi-square tests were used to compare categorical data and independent sample *t* tests to compare continuous variables. *BMI* body mass index, *CRP* C-reactive protein, *BASDAI* bath ankylosing spondylitis disease activity index (range, 1–10), *MASES* the maastricht ankylosing spondylitis enthesitis score (range, 1–13), *BASMI* bath ankylosing spondylitis metrology index, *BASFI* bath ankylosing spondylitis functional index (range, 0–10), *BAS-G* bath ankylosing spondylitis patient global score (range, 0–10), *HAQ* health assessment questionnaire (range, 0–4), *NSAIDs * non-steroidal anti-inflammatory drugs), *DMARDs* disease-modifying anti-rheumatic drugs

### SQOL and HRQOL

A similar pattern was seen for the comparisons of SQOL and HRQOL between patients reporting that their health status had little/no effect versus a large effect on sexual activity at the baseline and 5-year follow-up, and the differences were also larger at the 5-year follow-up (Table 3). At the 5-year follow-up, patients reporting that their health status had a large negative effect on sexual activity had considerably worse SQOL, as measured by the total score (64.6 [12.6] vs. 79.9 [8.6]) and the subcategories (all *p* < 0.001). Patients who perceived that their health status had a large effect on sexual activity also had worse HRQOL, as measured by the SF-36 PCS (33.5 [8.4] vs. 53.9 [9.1]; *p* < 0.001), SF-36 MCS (42.2 [10.5] vs. 50.0 [8.8]; *p* < 0.001), all eight SF-36 domains, and 15D (0.73 [0.09] vs. 0.89 [0.13]; *p* < 0.001).

**Table 3:** Sexual quality of life and health-related quality of life in patients with axial spondyloarthritis reporting that their health status had little/no effect (*n* = 200) versus a large effect (*n* = 44) at the baseline and 5-year follow-up

	Little/no effect baseline	Large effect baseline	*p* value	Little/no effect 5-year	Large effect 5-year	*p* value
Sexual quality of life						
SQOL sum	78.0 (10.6)	69.4 (13.4)	< 0.001	79.9 (88.6)	64.6 (12.6)	< 0.001
Psychosexual feelings	34.6 (7.9)	28.7 (9.1)	0.001	36.1 (6.2)	24.5 (9.2)	< 0.001
Sexual and relationship satisfaction	12.0 (5.6)	13.4 (4.6)	0.099	11.5 (5.5)	16.6 (5.7)	< 0.001
Self-worthlessness	15.9 (3.2)	13.7 (4.2)	0.002	16.1 (2.7)	12.1 (4.1)	< 0.001
Sexual repression	15.7 (3.3)	13.8 (4.2)	0.006	16.1 (2.7)	11.5 (4.4)	< 0.001
Health-related quality of life						
SF-36						
Mental health	78.6 (13.5)	75.2 (15.2)	0.176	80.2 (12.9)	69.7 (16.0)	< 0.001
Vitality	50.4 (19.9)	39.1 (20.6)	0.002	53.9 (20.6)	30.3 (17.7)	< 0.001
Bodily pain	50.5 (20.5)	42.7 (19.1)	0.019	58.5 (20.5)	37.2 (17.0)	< 0.001
General health	57.2 (20.9)	45.6 (21.6)	0.002	61.6 (21.6)	37.7 (16.6)	< 0.001
Social function	77.4 (21.4)	69.6 (22.6)	0.040	81.3 (20.2)	59.1 (22.8)	< 0.001
Physical function	77.4 (18.0)	61.4 (21.2)	< 0.001	80.6 (18.3)	55.8 (24.3)	< 0.001
Role limitation physical	49.0 (42.2)	29.0 (33.6)	0.001	59.5 (42.8)	28.9 (37.5)	< 0.001
Role limitation emotional	78.6 (36.0)	56.1 (44.2)	0.003	79.8 (35%)	53.8 (43.3%)	< 0.001
PCS	41.1 (9.6)	35.2 (8.0)	< 0.001	53.9 (9.1)	33.5 (8.4)	< 0.001
MCS	49.4 (9.0)	45.8 (11.3)	0.060	50.0 (8.8)	42.2 (10.5)	< 0.001
15D	0.87 (0.09)	0.78 (0.09)	< 0.001	0.89 (0.13)	0.73 (0.09)	< 0.001

The independent sample *t* test was used to compare between groups at the baseline and 5-year follow-up. The score for the 15D ranges from 0.0 (being dead) to 1.00 (no problems on any dimension). The score for the SF-36 ranges from 0 to 100 where 100 indicates a high HRQOL. SQOC = sexual quality of life, PCS = physical component summary, MCS = mental component summary

#### Baseline characteristics associated with a large negative effect of health status on sexual activity at the 5-year follow-up in the adjusted analyses

In the block-wise logistic regression analyses, old age and current use of NSAIDs at the baseline were significantly associated with a large negative effect of health status on sexual activity at the 5-year follow-up across all steps in the block-wise analyses (Table 4). In the analysis that included independent variables for the sixth and final step, the following baseline variables were found to be independently associated with a large negative effect of health status on sexual activity at the 5-year follow-up: old age (odds ratio [OR], 2.30; 95% confidence interval [CI] 1.00, 5.26); current use of NSAIDs (OR, 1.12; 95% CI 1.02, 1.22); and low SQOL (OR, 0.95; 95% CI 0.91, 0.99). In the model that included all independent variables in the final step, these variables explained 38% of the variance of the dependent variable. The same pattern of associations persisted after adjusting for the baseline perception that health status had little/no negative effect on sexual activity.

**Table 4:** Baseline demographic and disease-related variables associated with 5-year follow-up responses on perception state of health having a large (negative? ) effect on sexual activity in axial spondyloarthritis patients, tested in block wise logistic regression analyses

	Block 1 OR (95% CI)	*p*-value	Block 1 and 2 OR (95% CI)	*p*-value	Block 1–3 OR (95% CI)	*p*-value	Block 1–4 OR (95% CI)	*p*-value	Block 1–5 OR (95%)	*p*-value	Block 1–6 OR (95% CI)	*p*-value
Demographic												
Age, years	1.06 (1.01, 1.10)	0.009	1.06 (1.01, 1.11)	0.024	1.05 (1.00, 1.11)	0.047	1.07 (1.01, 1.13)	0.017	1.09 (1.03, 1.16)	0.005	1.10 (1.03, 1.17)	0.004
Woman	0.61 (0.23, 1.63)	0.323	0.43 (0.15, 1.24)	0.118	0.43 (0.15, 1.24)	0.118	0.53 (0.18, 1.55)	0.245	0.32 (0.10, 1.02)	0.053	0.34 (0.10, 1.12)	0.076
Married\cohabiting	1.18 (0.36, 3.87)	0.781	0.98 (0.28, 3.40)	0.978	0.98 (0.28, 3.41)	0.978	1.01 (0.27, 3.79)	0.992	0.69 (0.18, 2.61)	0.572	0.66 (0.17, 2.52)	0.540
Exercise > 1 h/week	0.56 (0.17, 1.79)	0.324	0.55 (0.16, 1.88)	0.334	0.59 (0.17, 2.06)	0.405	0.60 (0.17, 2.07)	0.420	0.83 (0.21, 3.36)	0.794	1.27 (0.28, 5.70)	0.755
BMI (kg/m^2^)	1.02 (0.92, 1.13)	0.690	1.02 (0.91, 1.13)	0.793	1.02 (0.91, 1.13)	0.785	1.02 (0.91,1.14)	0.775	1.02 (0.90, 1.15)	0.741	1.02 (0.90, 1.16)	0.727
Education > 13 years	0.98 (0.42, 2.32)	0.966	1.21 (0.45, 2.98)	0.683	1.17 (0.67, 2.92)	0.741	0.88 (0.33, 2.38)	0.803	0.83 (0.29, 2.39)	0.727	0.86 (0.29, 2.51)	0.781
Clinical measures												
CRP (mg/dl)			0.99 (0.95, 1.03)	0.506	0.99 (0.94, 1.03)	0.529	0.99 (0.95, 1.03)	0.612	0.97 (0.93, 1.01)	0.180	0.98 (0.93, 1.03)	0.353
BASDAI			1.22 (0.92, 1.63)	0.166	1.23 (0.92, 1.64)	0.155	1.25 (0.92, 1.68)	0.145	1.10 (0.79, 7.12)	0.576	0.98 (0.68, 1.41)	0.894
Health status												
HAQ (range 0–3)			1.87 (0.53, 6.58)	0.328	1.80 (0.50, 6.42)	0.368	1.42 (0.38, 5.32)	0.597	1.78 (0.45, 7.12)	0.412	1.01 (0.22, 4.64)	0.990
Damage												
BASMI			0.92 (0.73, 1.15)	0.469	0.92 (0.73, 1.15)	0.454	0.89 (0.69, 1.15)	0.383	0.88 (0.63, 1.10)	0.208	0.81 (0.61, 1.07)	0.138
Comorbidity Total score for comorbidity (range 0–10)					1.13 (0.65, 1.98)	0.666	1.39 (0.76, 2.51)	0.283	1.20 (0.61, 2.33)	0.602	1.26 (0.64, 2.4)	0.502
Current treatment												
NSAIDs							3.44 (1.26, 9.4)	0.016	3.48 (1.20, 10.13)	0.022	3.77 (1.27, 11.17)	0.017
sDMARD							1.30 (0.19, 8.70)	0.786	1.2 (0.16, 9.29)	0.861	1.03 (0.12, 8.61)	0.979
bDMARD							2.45 (0.85, 7.03)	0.097	1.88 (0.61, 5.73)	0.270	2.12 (0.67, 6.79)	0.204
Quality of Life												
SQOL									0.93 (0.89, 0.97)	0.001	0.94 (0.90, 0.98)	0.002
SF-36-PCS											0.93 (0.86, 1.01)	0.080
SF-36-MCS											0.97 (0.92, 1.03)	0.315

Range 0–100, where 100 indicates a high health-related quality of life (HRQoL). OR, odds ratio; *CI* confidence interval; *BMI *body mass index; *BASDAI *bath ankylosing spondylitis disease activity index; *BASMI* bath ankylosing spondylitis metrology index; *BASFI *bath ankylosing spondylitis functional index; *BAS-G* bath ankylosing spondylitis patients global score; *HAQ* health assessment questionnaire; *SF-36* 36-item short form health survey; *PCS* physical component summary; *MCS *mental component summary; *NSAID *nonsteroidal anti-inflammatory drug; *sDMARD *synthetic disease-modifying anti-rheumatic drug; *bDMARD *biological disease-modifying anti-rheumatic drug. SQOL

## Discussion

We explored the perceived long-term effects of health status on sexual activity in patients with ax-SpA and identified the predictors associated with SQOL after 5 years. At the 5-year follow-up, 18% of the patients reported that their health status had large negative effects on their sexual activity. This was the same percentage as at the baseline. In the comparison between patients according to their perception of the effects of health status on sexual activity, those who perceived that their health status had a large effect were older, were less likely to be employed, exercised less, had more comorbidities and higher disease activity, and reported lower HRQOL and SQOL scores. The baseline predictors of a large negative effect of health status on sexual activity at 5 years were old age and low SQOL at the baseline. Although disease control was better after 5 years, the patients reported more comorbidities.

The better disease control along with lower disease activity parameters and greater use of bDMARDs in these patients most likely reflect the targeted approach and more active use of biological treatments during the 5-years of follow-up [51, 52]. The treatment approach for patients with ax-SpA changed during the 5 years of the study, when there was an increasing focus on the combination of non-pharmacological and pharmacological treatments [53, 54] The current treatment approach aims to be holistic and individualized, and to consider the disease symptoms as well as other aspects such as comorbidities, medication, and QOL [51, 52]. The main improvement in clinical outcome, both reducing inflammation and patient perception of the disease, seen in ax-SpA patients with improved QOL over the last decades is predominantly thought to be related to treatment with bDMARDs [35, 55]. Thus, there is also reason to believe that increased use of bDMARDs as shown in our study do also impacts sexual activity positively.

The number of patients reporting that their health status had a large negative effect on sexual activity were stable over the 5-years period, although a few patients changed from perceiving their health status as having little/no effect and vice versa. In general, those patients who perceived that their state of health had a large negative effect reported a less healthy lifestyle and greater disease activity. Other studies have reported associations between disease activity and lack of physical activity with a negative effect on sexual activity [40, 41]. For patients whose perception changed from a negative effect of health status to little/no effect on sexual activity, this change may have reflected the use of a more holistic and targeted treatment approach during at the 5-year follow-up, as noted above [53, 54]. For those whose perceptions were in the opposite direction, we speculate that an increased number of comorbidities may have negatively influenced their perception of how health status affected their sexual activity.

The patients who perceived that their health status had a large negative effect on sexual activity reported lower HRQOL in both HRQOL questionnaires [42, 44, 45, 47] used in the present study. Having more comorbidities and greater disease activity, such as restricted physical and mental functioning, tend to negatively influence HRQOL [6, 40]. One could argue that the topic covered by question 15 in the 15D questionnaire is also reflected in some of the questions in the self-reported measures of disease activity, damage, and health status, such as the BAS-G, BASDAI, BAS-G, and HAQ [56, 57] and the HRQOL measures [42, 45, 47]. On the other hand, question 15 focuses explicitly on sexual activity, which is not covered in the other questionnaires [42].

The patients who reported that their health status had a large negative effect on sexual activity also reported lower SQOL. The phenomenon (sexual activity) included in question 15 in 15D covers some of the same aspects of SQOL [42]. However, sexual activity and SQOL are also distinct. Sexual activity focuses on reproduction and the expression of sexuality as the physical dimension of QOL and can be affected by many characteristics, such as interpersonal relationships, socio-cultural conditions, environmental factors, and how sexually active a person or couple is. Sexual activity is also influenced by a person’s physical and mental health and hormonal status [58]. SQOL focuses more on the relationship between sexual dysfunction and QOL, and is defined as the individual’s subjective evaluation of the positive and negative aspects of his/her sexual relationship and the subsequent affective response to this evaluation [9, 10].

Only a few baseline variables were found to be independently associated with a large negative effect of health status on sexual activity at the 5-year follow-up: old age, current use of NSAIDs, and low SQOL. In the final step of the analysis, these variables explained 38% of the variance of the dependent variable. NSAIDs are considered as a first-line drug in the treatment of ax-SpA. The use of NSAIDs to ameliorate disease activity, including pain, stiffness, fatigue, and difficulties sleeping [15], may lead to physical difficulties when having sex [12, 14]. Sexual activity is considered a part of a good SQOL and is influenced by personal characteristics, relationships with a partner and family, socio-cultural conditions, and one’s own physical and mental health [58]. Sexual activity may also change in different phases of life and with age. In the present study, old age was independently associated with a large negative effect of health status on sexual activity. There is little consensus in the literature about sexual activity in older age groups, which appears to be heterogeneous, and it is difficult to compare studies because the relationships between sexual activity, SQOL, and older age are multi-determined and exhibit larger individual variability [59].

### Strengths and limitations

One strength of the present study is the unselected population because most patients attending the outpatient clinics were invited and included, which increases the validity of the study. Another strength is that most of the data were collected from routine practice, which means that the findings may be relevant to patients and health professionals. The questionnaires have been validated, which further increases the validity of the study. Finally, the longitudinal nature of the study with a large sample size showed consistent associations between health status and a large negative impact on SQOL.

A limitation of this ax-SpA study is the uneven number of women (30%) and men (70%) [60]. In ax-SpA which includes both the radiographic and the non-radiographic phenotype of ax-SpA, as also reflected in the ASAS criteria, the gender distribution is more equal as compared with the radiographic phenotype of ax-SpA with more men having this phenotype [60]. Unfortunately, a large proportion of patients did not have x-rays performed thus we can not provide information on radiographic status. Thus, there is reason to believe that a majority of our included patients actually had radiographic ax-SpA which would explain the gender distribution, and we could argue that it might be hard to identify the differences on how health status influence on sexual activity in women and men respectively. Furthermore, our patients were included consecutively and are representative of the patients coming to the out-patients clinic in the time-period of inclusion. Another limitation is that the data were collected at only two time points. Data for the 140 patients who were not invited to the 5-year follow-up or panel attrition may have contributed to different results. Previous studies have shown that panel attrition tends to involve less healthy patients [61]. However, we identified small differences in the baseline characteristics between those who attended the follow-up and those who were lost to follow-up. The given date for stopping the invitations at the 5-year follow-up at one of the hospitals also increased the possibility of random panel attrition.

The strong association between the self-reported outcome measures that reflected disease activity and burden with the perception that health status had a large negative effect on sexual activity, measured by generic questionnaires, can be considered as both a strength and a limitation. It is possible that the disease-specific measures captured the actual disease burden of ax-SpA; however, some of the items included in both the disease-specific and generic questionnaires are similar and may therefore correlate with each other. Another limitation is that the number of independent variables for some of the multivariable logistic regression models was high relative to the sample size. Finally, the use of one single item to measure perceived effect of health status on sexual activity and the outcome five-point Likert scale reduced to two, might reduce sensitivity, specificity and attenuating of the outcomes. However, we choose to use a more anchor based approach dichotomizing the dependent variable to make the analysis easier to interpret [62]. Such a dichotomization of the dependent variables is used in other studies as well [40, 41], and other more complementary and emotional approaches to sexuality is covered by the SQOL-measure used in the present study.

## Conclusion

At the 5-year follow-up, 18% of the patients with ax-SpA reported that their health status had large negative effects on their sexual activity; this was the same percentage as at the baseline. Patients who perceived that their health status had a large effect on sexual activity at the 5-year follow-up were older, less likely to be employed, exercised less, had more comorbidities and higher disease activity, and reported lower HRQOL and SQOL. Older age and low SQOL at the baseline predicted a large self-reported negative effect of health status on sexual activity by patients with ax-SpA. Our results suggest the importance of individualized treatment and disease control of patients with ax-SpA. To assist more patients with ax-SpA to maintain sexual activity and good HRQOL and SQOL, health-care professionals could focus on older patients, and encourage good disease control.

## Data Availability

The datasets used and/or analysed during the current study are available from the corresponding author on reasonable request.
